# Audit of Antibiotic Practices: An Experience from a Tertiary Referral Center

**DOI:** 10.5005/jp-journals-10071-23104

**Published:** 2019-01

**Authors:** Ritu Singh, Afzal Azim, Mohan Gurjar, Banani Poddar, Arvind K Baronia

**Affiliations:** 1-5 Department of Critical Care Medicine, Sanjay Gandhi Postgraduate Institute of Medical Sciences, Lucknow, Uttar Pradesh, India

**Keywords:** Antibiotic, Audit, De-escalation

## Abstract

**Aims:**

To estimate the prevalence of antibiotic de-escalation at admission in patients referred to a tertiary hospital in India. The secondary outcomes were the adequacy of empirical antibiotic therapy and culture positivity rates in the de-escalated group.

**Materials and methods:**

A prospective observational study, in a 20-bedded intensive care unit (ICU) of tertiary care hospital. Patients >18 years, surviving > 48 hours, were included (June– December 2017). Demographic data, previous cultures, and antibiotics from other hospitals, laboratory parameters in the first 24 hours, and severity of illness were noted. Changes made in antibiotic therapy within 48 hours were recorded. Patients were analyzed into three groups: “No change”–empiric therapy was maintained, “Escalation”–switch to or addition of an antibiotic with a broader spectrum, and “De-escalation”–switch to or interruption of a drug class.

**Results:**

The total number of patients eligible was 75. The mean age of the population is 43.38 (SD + 3.4) and groups were comparable in terms of mean sequential organ failure assessment score (SOFA) and acute physiology, age, chronic health evaluation (APACHE) 2. The prevalence of de-escalation was 60% at admission. The escalation group consisted of 24%. Sixteen percent patients belonged to no change group. Results showed that 38% of patients were on carbapenems, dual gram negative was given to 26%, and empirical methicillin-resistant *staphylococcus aureus* (MRSA) coverage was 28% on admission.

**Conclusion:**

Our study aims to provide data about actual practices in the Indian scenario. It highlights the generous use of high-end antibiotics in the community. Indian practices are far cry from theoretical teaching and western data. The need for antibiotic stewardship program in our country for both public and private health sectors is the need of the hour.

**How to cite this article:**

Singh R, Azim A, Gurjar M, Poddar B, Baronia AK. Audit of Antibiotic Practices: An Experience from a Tertiary Referral Center. Indian Journal of Critical Care Medicine, January 2019;23(1):7-10.

## INTRODUCTION

Antibiotic resistance is a globally emerging problem.^1^ Developing countries like ours have to face dual problem of access and excess of antibiotics. Even though evidence supports the use of broad-spectrum antibiotics initially to reduce mortality, its indiscriminate use has led to the emergence of resistance.^2^ De-escalation is an important component of antibiotic stewardship.^3^ The definition of de-escalation encompasses two key features. First, is to narrow the spectrum of antimicrobial coverage depending on clinical response, culture results, and susceptibilities of the pathogens identified; and second, is to stop antimicrobial treatment if no infection is established.^4^ Need for de-escalation has already been highlighted well in literature. But data about actual practices is lacking especially in our country. Rates of de-escalation range from about 10% in studies of clinical practice to about 70% in specifically designed trials.^5^ Estimating the prevalence of de-escalation in Indian ICUs will give us the true picture of what we actually do. Identifying the problems and challenges faced in adopting the practice de-escalation, may help guide further research especially in the context of Indian scenarios. We conducted this study to analyze the appropriateness of antibiotic therapy in patients referred to our center after prior hospitalizations.

## MATERIALS AND METHODS

It is a prospective observational study carried out in a 20-bedded general purpose ICU of a tertiary care center in Northern India. Prior approval from the local ethics committee was taken before conducting this study. The study period was from June 2017 to December 2017.

All patients above the age of 18 who survived >48 hours after ICU admissions were included. Demographic data like age, sex, comorbidities, duration of previous hospital stay, previous cultures and antibiotics from other hospitals; laboratory parameters (including biomarker for infection) in the first 24 hours, and severity of illness (admission APACHE 2, SOFA) were noted. Changes made in antibiotic therapy in the first 48 hours were recorded. Subsequently, patients were analyzed into three groups. Group 1: No change. It was defined as a continuation of antibiotic therapy with which patient was transferred. Group 2: Escalation group. It was defined as the patient population where a switch to or addition of an antibiotic with a broader was done. Group 3: De-escalation group. This group is defined as a patient population where there’s a switch to, or interruption of a drug class was done, resulting in a less broad spectrum of coverage.^4^ Sepsis or septic shock was defined as per the recent consensus definition in Sepsis 3.^6^

## RESULTS

**Graph 1:**
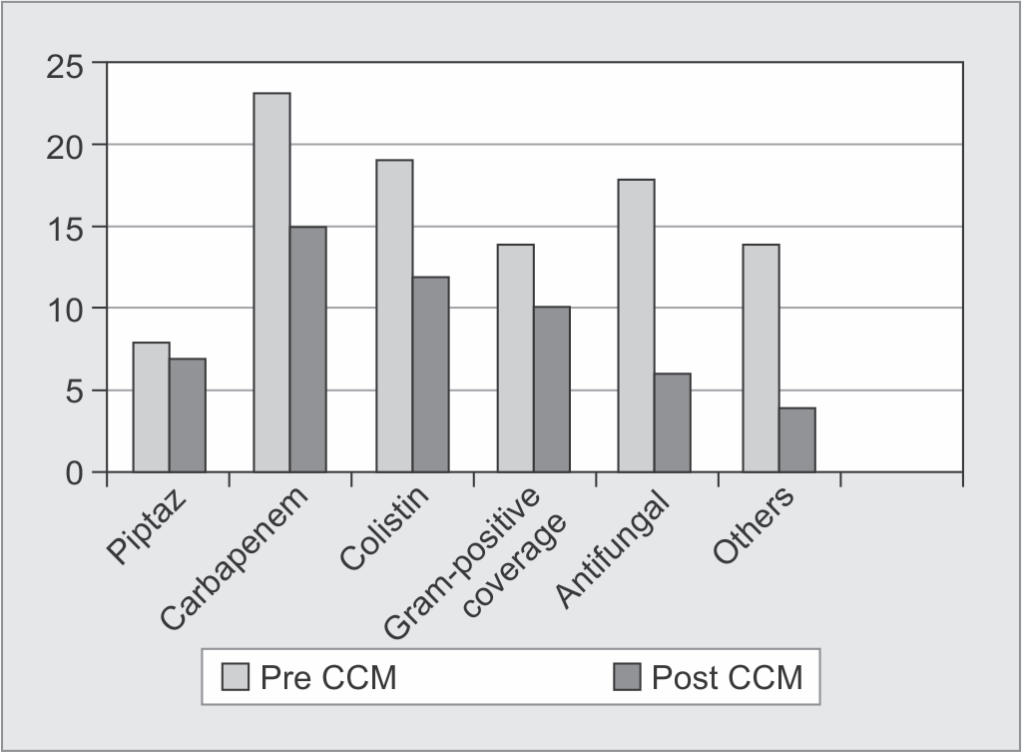
Antibiotic change in de-escalated group

A total of 75 patients were enrolled over a 6-month period. The characteristics observed among the groups are shown in Table 1. The mean age of the population was 43.38 (SD + 3.4). The disease severity was comparable in terms of mean SOFA and APACHE 2. The prevalence of deescalation on admission to our ICU was 60% (Graph 1). 45% of the de-escalated patients had an available previous culture and a mean stay of 13.72 days in the previous hospitals. Our de-escalation decisions were based on these cultures and clinical judgment of the intensivist. Our evaluation of admission culture in the de-escalated group further supported the decision, as all culture were sterile in 68.8% of patients (Table 2). 20% of the de-escalated group patients had ET/ TT culture positive.

**Graph 2:**
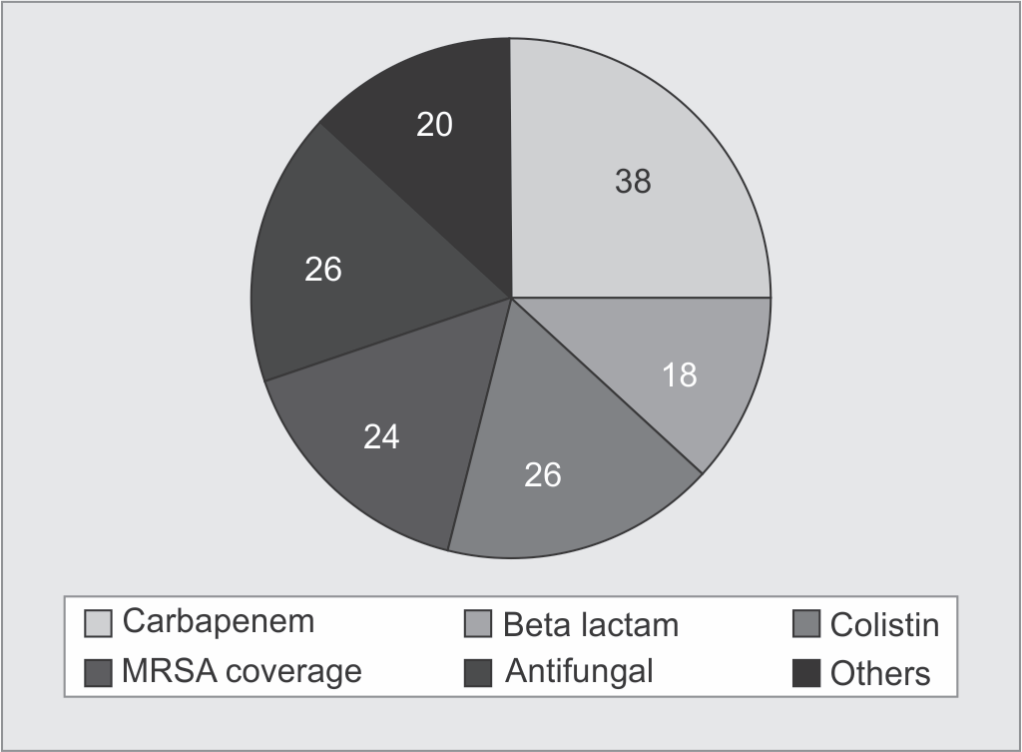
Antibiotics on admission to ICU (%age)

The escalation group consisted of 24% of patients who had a mean hospital stay of 7 days in the previous hospital, with previous culture positivity of 18%. Most of these patient’ s admission culture showed positive blood cultures (15%) and antibiotics were revised as per sensitivity. Sixteen percent patients belonged to ‘No Change’ group, and antibiotics were continued in these group due to lack of available cultures. The antibiotics on admission are reviewed in Graph 2. It showed that 38% of patients were on carbapenem. Dual gram-negative (colistin + carbapenem) were given to 26% patients. Empirical MRSA coverage was given to 28% patients. Use of beta-lactam group was only in 18%. Empirical antifungals were used in 26% patients. Other group, which included atypical coverage, was 20%.

**Table 1:** Baseline characteristics of patients (n = 75)

*Characteristics*	*De-escalation (n = 45)*	*Escalation (n = 18)*	*No change (n = 12)*	*p value*
Age (mean)	45.76	39.88	43.42	0.49
Male	28	10	8	
Comorbidities HTN Diabetes Postoperative	10 9 0	0 6 4	8 4 0	
Mean APACHE 2	15.2	14.94	11.62	0.38
Mean SOFA	8.8	10.23	9.62	0.49
Previous hospital exposure (median days)	13.72	7.24	7.70	0.31
Diagnosis at admission Neurological Respiratory Gastrointestinal Cardiovascular Renal Infectious	8 20 9 6 8 8	3 9 11 7 9 5	2 4 5 3 7 2	
Culture at admission present	28 (45)	11 (18)	7 (12)	
No of empirical Antibiotic 0–1	4	5	2	
No of empirical Antibiotic 2	9	11	3	
No of empirical antibiotic 3 or more	32	2	7	

## DISCUSSION

The alarming rate of indiscriminate use of broadspectrum antibiotics in the community (especially in third world countries, like India) is a matter of concern. Dhruv et al. in their study have talked much about the prevalence of multi-drug-resistant (MDR)-organisms in India and how they differ from western data. It is well highlighted in the literature the high prevalence of gram-negative organisms (76–93%) in India and their resistance pattern.^7^ Most clinicians advocate de-escalation, but its actual day-to-day practice is a far cry from reality. Hence the need of the hour is the development of antibiotic stewardship programs based on local epidemiological data as previously supported by the literature. But for an effective stewardship program not only the local antibiogram is needed but an audit of practices is also essential. Carbapenems, which were initially used sparingly, are now being widely used in community hospitals and nursing homes all across northern India. Our study showed 38% of referred patients were on carbapenems on admission to our ICU. We recorded a gradual change of practice in the use of empirical antibiotics from β-lactam to carbapenem group pointing a trend towards the use of higher end empirical antibiotics which out much clinical/microbiological data. Lack of protocolized approach of antibiotic use and awareness about de-escalationhas led to continued use of broad spectrum as well as more number of antibiotics. Seventy-one percent of patients were on 3 or more antibiotics on admission (Table 1). An Indian study done in 2015 highlighted this trend and reported carbapemens to be the most common antibiotic used empirically.^8^ Indiscriminate use of many broad-spectrum antibiotics promotes the emergence of MDR organisms and also limits the availability of antibiotics in the armamentarium of tertiary ICUs like ours. A step-up approach for antibiotics depending upon the level of ICU/ward should be outlined. Ahmed et al. have laid down a simple algorithm for choosing antibiotic therapy for life-threatening infection and MDR organisms.^9^ The compliance of such algorithm in the community is still questionable.

**Table 2:** Clinical features/culture of de-escalation group (n = 45)

*Clinical features*	*No.of patients*
No shock at admission	23
TLC <11,000	16
Admission culture	
ET/TT culture	9
Blood culture	3
Urine culture	2
All cultures sterile	31

Amongst the patients studied, based on clinical features on admission our de-escalation rate was 60%. Sixty-eight per cent of the follow up cultures at admission in the de-escalation group were negative (ET, blood and urine) as shown in Table 2. Twenty percent had ET cultures positive but were mostly colonizers (clinical pulmonary infection score <6) and 6% had cultures positive (mostly blood) which required stepping up the treatment.

Realizing the theoretical importance of de-escalation is not the only measure. Awareness and audit of practices is the next important step towards de-escalation.^10^ Our audit study highlights the practices in several districts of Uttar Pradesh and neighboring states and alarming use of a wider range of empirical antibiotics–both in terms of spectrum and no of antibiotics. A concept of antibiotic “Time Out” was defined in a study by Daniel Markley.^11^ Time out is meant to prompt the clinician to perform a reassessment of the need and the spectrum of antibiotics as diagnostic data and clinical picture of the patient emerges. They recommended that reassessment should be performed every 48 hours after initiation of antibiotics. Various laboratory parameters to guide de-escalation have also been defined in the literature. As seen by our study its actual adherence in clinical practice is a matter of concern. Barriers to adherence should be identified early and local step up approach for use of antibiotics be outlined by national agencies. Studies have found paradoxical practices of broadening of antibiotics instead of de-escalation on clinical improvement.^12^ Limited availability of diagnostic tests at various centers in India is one of the major barriers to de-escalation. Definitive diagnosisis usually not made and treatment is based solely on the clinical judgment. There is a feeling of insecurity in de-escalating antibiotics when there is clinical improvement.^13^ Rapid diagnostic tests like matrix-assisted laser desorption ionization (MALDI)-TOF, beta-D glucan, Procalcitonin should be widely available to encourage de-escalation. Another barrier is the availability of low-quality evidence to guide management. Most recommendations about de-escalation were based on level III evidence, and only 15% were based on the highest quality evidence (data from randomized controlled trials).^14^ Hence, literature which supports de-escalation should be highlighted and practiced. One of the studies that explored the practical application of de-escalation, collected data from 113 ICU and meropenem prescriptions were evaluated. De-escalation was defined as the administration of an antibiotic with a narrower spectrum within 3 days of the start of meropenem. The study found a trend toward a lower mortality rate (7% *vs.* 21%) in patients who had been de-escalated.^15^ A retrospective study was done in 2015 by Lee et al. in community-acquired bacteremia, had a de-escalation rate of 45% and was protective for mortality. Most studies which talk about de-escalation either report increase mortality or increase length of hospital stay.^16,17^ High quality of evidence and more studies are required to boost our theoretical knowledge of de-escalation practices.

## CONCLUSION

Our study gives data about the trends in antibiotic use and de-escalation rates in the Indian scenario. It focuses on the use of antibiotics in the community who were referred from various hospitals and nursing homes to our tertiary level ICU. Our study also highlighted the urgent need of a step up approach of the antibiotic stewardship program in our country and the barriers to de-escalation in the Indian scenario. A larger study establishing the need a protocolized approach and improve the quality of data for de-escalation strategies is the need of the hour.
